# Posterior cervical spine surgery epidemiology and complications: a large retrospective case series

**DOI:** 10.31744/einstein_journal/2025AO1830

**Published:** 2025-09-08

**Authors:** Rodrigo Cozar Silva, Fellipe de Paula, Rômulo Augusto Andrade-Almeida, Andrei Joaquim

**Affiliations:** 1 Universidade Estadual de Campinas Campinas SP Brazil Universidade Estadual de Campinas, Campinas, SP, Brazil.; 2 The University of Texas MD Anderson Cancer Center Houston Texas United States The University of Texas MD Anderson Cancer Center, Houston, Texas, United States.

**Keywords:** Cervical spine, Surgical procedures, Epidemiology, Postoperative complications

## Abstract

**Objective:**

Posterior cervical spine surgery is used to decompress and/or stabilize the spine for the treatment of various spinal diseases. The aim of this study was to evaluate the clinical characteristics, surgical indications, and complications of patients who underwent posterior cervical spine surgery at a tertiary center.

**Methods:**

This retrospective cohort/case series study included data from patients who underwent posterior cervical spine surgery at a tertiary hospital to treat different cervical diseases.

**Results:**

A total of 161 patients were included. One hundred six (65.8%) patients were men, and mean age was 45.1 years. Patients with neoplastic diseases had the lowest mean age, whereas those with traumatic and degenerative diseases had the highest (p<0.001). Thirty-six patients (22.3%) experienced at least one complication. Serious adverse effects were infrequent despite six deaths (3.7%), and among the non-serious complications, surgical site infection (6.2%) and the need for late reoperation (4.3%) were the most common. No specific patient characteristics were associated with complications; however, a trend toward complications in urgent procedures was noted (p=0.085).

**Conclusion:**

Posterior cervical spine surgery was more common in men, and patients with degenerative diseases had a higher average age. There was no statistically significant association between complications and patient characteristics, with a trend toward more clinical complications during urgent procedures. Serious complications were infrequent in elective procedures; however, a small risk of death was noted, particularly in patients with trauma-related cervical spine injuries. Understanding the epidemiology and complications is fundamental for preoperative counseling and the prevention of complications.

## Highlights

■Posterior cervical spine surgery showed an overall complication rate of 22.3%, mainly surgery-related.

■Surgical site infection (6.2%) and late reoperation (4.3%) were the most common complications.

■The average patient age varied by etiology, being the lowest in neoplastic cases (34.4 years) and the highest in degenerative cases (55.7 years).

■No statistically significant associations were found between complications and patient characteristics, except for a trend toward more complications in urgent surgeries (p=0.085).

■Mortality was 3.7%, mainly among patients with traumatic cervical injuries and severe preoperative spinal cord dysfunction.

## In Brief

This retrospective study evaluated the data of 161 patients who underwent posterior cervical spine surgery over 10 years. Complications occurred in 22.3% of the patients, with surgical site infection and late reoperation being the most frequent. No patientrelated factors were significantly associated with complications, although urgent surgery showed a trend toward higher complication rates. The procedure was safe overall, with serious complications being uncommon and mainly related to trauma.

## Graphical abstract

**Figure:**
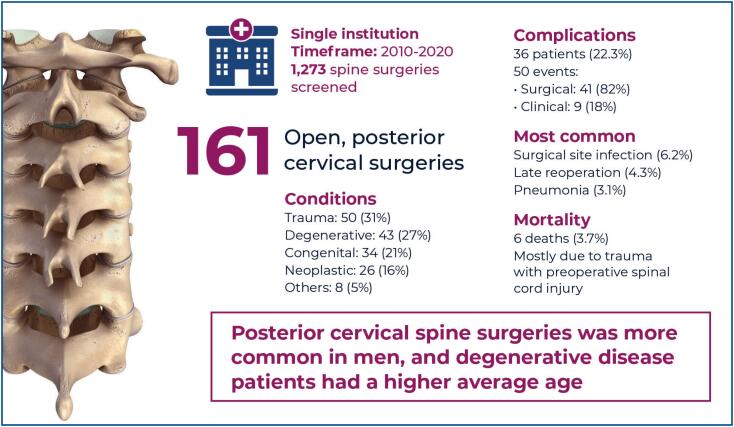

## INTRODUCTION

The cervical spine can be impacted by multiple conditions of varying etiologies. Traumatic injuries can occur, causing fractures, hematomas, and/or disruption of the supporting elements of the spine. The cervical spine can also harbor benign and malignant neoplasms that cause compression and destruction of the surrounding normal tissues. Degenerative conditions may be present, particularly in older patients, causing symptoms that are related to compression of the spinal cord or nerve roots. Other conditions include congenital malformations, infections, and inflammatory diseases. Severe pathologies involving critical neural compression, mechanical instability, severe refractory pain, or deformity usually require surgery.^(1)^

Surgical approaches depend on the characteristics of the disease and patient, as well as the surgeon´s preferences.^(2,3)^ Currently, the most common surgical approaches for the cervical spine are anterior and posterior, which have different indications and complications.^(4-8)^ Some techniques and their specific indications are in consensus among surgeons, whereas others are not.^(9,10)^

Various authors have reported their experiences or published literature reviews of complications related to open posterior cervical procedures.^(6-8,11,12)^ Studies have suggested that the posterior approach is associated with higher hospital expenses and length of stay.^(7,8)^ This approach has also been associated with complications in 15%–25% of cases, such as acute blood loss anemia, surgical site infection, C5 palsy, and incidental durotomy.^(11)^ The complication profile varies according to patient and disease characteristics.^(11)^ Nevertheless, open posterior cervical spine surgery (PCSS) remains an important tool in the armamentarium of spine surgeons.^(13)^

## OBJECTIVE

This study aimed to report our experience with patients at a tertiary hospital who underwent open posterior cervical spine surgery to manage cervical or occipitocervical conditions, with a focus on the complication profiles.

## METHODS

### Study design, population, and ethics

This retrospective cohort study was approved by the local institutional review board of the *Faculdade de Ciencias Medicas da Universidade Estadual de Campinas* (CAAE: 47576821.0.0000.5404; #4881515), which waived the requirement for written informed consent. A database of patients who underwent any surgical spinal procedure was screened for eligibility criteria. All patients of any age who underwent pure (no combined approaches), open PCSS performed by a senior surgeon at a tertiary university hospital between 2010 and 2020 were identified and included for evaluation.

### Data and statistical analysis

Retrospective data collected from electronic records included sex, age, etiology, spinal segment affected, type of surgery (elective vs. emergency), date of the last clinical follow-up, and presence of clinical or surgery-related complications.

Patients were grouped according to etiology as degenerative, traumatic, congenital, neoplastic, or other (infectious or inflammatory). Cases were classified anatomically according to the vertebral level as follows: upper cervical (occipital-C2), subaxial (C3-C7), cervical-thoracic, and mixed (more than one category). Clinical complications of interest included pneumonia, urinary tract infection (UTI), deep vein thrombosis (DVT), and pulmonary thromboembolism (PTE). Surgical complications included site infection, hematoma, cerebrospinal fluid leakage, wound dehiscence, neurological impairment, early reoperation (during the same hospitalization), late reoperation (during another hospitalization), instrument breakage, and pseudarthrosis. Patient deaths were further subcategorized as surgical or nonsurgical.

Data were analyzed using Jamovi statistical software (Jamovi v.2.3.28 [Sydney, Australia]). Continuous variables are summarized as mean and standard deviation for normal distribution or as median and interquartile range for non-normal distribution. Categorical variable proportions are summarized as absolute and relative values.

The tests used for the comparative analyses were chosen and reported according to the variables and population characteristics. An alpha value of < 5% (p<0.05) was adopted to reject the null hypothesis.

## RESULTS

The data from a total of 1,273 surgeries were screened, including surgeries of any spinal segment and approach. Of these, 161 (12.6%) met the eligibility criteria and were included in the analysis. The demographic and surgical data from this cohort are presented in table 1.

**Table 1:** Patient demographics and baseline characteristics by etiological group

Variable		Etiological Group					Subtotal n (%)
		Traumatic n=50 (31%)	Degenerative n=43 (27%)	Congenital n=34 (21%)	Neoplastic n=26 (16%)	Others n=8 (5%)	
Age range, n (%)		-	-	-	-	-	-
	0 to 17 years	2 (4.0)	1 (2.3)	2 (5.9)	7 (26.9)	0 (0.0)	12 (7.5)
	18 to 59 years	36 (72.0)	23 (53.5)	27 (79.4)	14 (53.8)	6 (75.0)	106 (65.8)
	60+ years	12 (24.0)	19 (44.2)	5 (14.7)	5 (19.2)	2 (25.0)	43 (26.7)
	Mean age, years	43.7	55.7	39.9	34.4	52.5	-
Sex, n (%)		-	-	-	-	-	-
	Male	44 (88.0)	31 (72.1)	15 (44.1)	12 (46.2)	4 (50.0)	106 (65.8)
	Female	6 (12.0)	12 (27.9)	19 (55.9)	14 (53.8)	4 (50.0)	55 (34.2)
Segment, n (%)		-	-	-	-	-	-
	Subaxial	30 (60.0)	33 (76.7)	1 (2.9)	8 (30.8)	3 (37.5)	75 (46.6)
	Upper cervical	15 (30.0)	2 (4.7)	31 (91.2)	7 (26.9)	2 (25.0)	57 (35.4)
	Mixed	2 (4.0)	7 (16.3)	2 (5.9)	8 (30.8)	3 (37.5)	22 (13.7)
	Cervico-thoracic	3 (6.0)	1 (2.3)	0 (0.0)	3 (11.5)	0 (0.0)	7 (4.3)
Surgery type, n (%)		-	-	-	-	-	-
	Elective	39 (78.0)	41 (95.3)	33 (97.1)	25 (96.2)	8 (100.0)	146 (90.7)
	Urgency	11 (22.0)	2 (4.7)	1 (2.9)	1 (3.8)	0 (0.0)	15 (9.3)

In the overall cohort, data showed a higher prevalence of male patients (n = 106, 65.8%), with a ratio of 1.92:1. In the groups "traumatic" and "degenerative" there was a male predominance of 88% and 72%, respectively. Conversely, females represented a larger proportion in the groups "congenital" (56%) and "neoplastic" (54%), although the difference was minimal. Mean age was 45.1 ± 17.9 years (95% confidence interval, 42.5–48.5 years). Degenerative cases presented a higher mean age (55.7 years) than that of the other etiologies, whereas patients with tumors had the lowest mean age (34.4 years).

Most surgeries were elective (n=146; 90.7%), with only 15 (9.3%) performed emergently. In addition, most emergency surgeries were performed for trauma-related injuries (11 patients, 73% of all emergency surgeries). Notably, when considering the anatomical site, the "traumatic" group demonstrated more frequent involvement of the subaxial cervical spine (n = 30; 60% of cases), followed by the upper cervical (n=15; 30% of cases); the latter being largely due to fracture of the odontoid process. Among the degenerative diseases, most cases were subaxial (33 cases, 77%), unlike congenital diseases, where most occurred in the upper cervical region (31 cases, 91%).

The patients were followed up for a mean period of 37.7 months (median, 25.6 months; range, one month–12 years), during which 36 (22.3%) patients presented with complications. Twenty-five patients had one complication, eight had two, and three patients had three, with a total of 50 events (clinical and surgical). Six patients (3.7%) died, including all those who had three complication events. Among these, four had associated severe spinal cord dysfunction due to spinal trauma, one woman with congenital craniocervical malformation had ventilatory intercurrence four weeks after surgery with tracheal collapse, and one had cerebral palsy with severe cervical myelopathy. The causes of death included septic shock (n=3), cardiorespiratory arrest (n=2), and pulmonary embolism (n=1). Interestingly, five of the six deceased patients had severe spinal cord dysfunction before surgery, which may indicate an inherent risk of increased death after surgery.

No correlations were found between the complications and sex, age, etiological group, or anatomical classification. Of the 50 complications in the 36 patients, nine (18%) were clinical and 41 (72%) were surgical, with no statistical significance across patient characteristics, except for surgery type (elective vs. urgent), with a trend towards more complications in urgent procedures (p=0.085) (Tables 2 and 3). A breakdown of the complications into surgical and clinical complications revealed that urgent procedures were significantly associated with surgical complications. No significant association was found between urgent procedures and clinical complications (Table 4).

**Table 2:** Association between patients’ characteristics and surgery complications

Characteristics		Patients with complications		χ^2^	p value
		Yes (n=36)	No (n=125)		
Age range, n (%)					
	0 to 17 years	3 (8.3)	9 (7.2)	0.463	0.794*
	18 to 59 years	22 (61.1)	84 (67.2)		
	60+ years	11 (30.6)	32 (25.6)		
Sex, n (%)					
	Male	21 (58.3)	85 (68.0)	1.16	0.281†
	Female	15 (41.7)	40 (32.0)		
Etiological group, n (%)					
	Traumatic	11 (30.6)	39 (31.2)	2.96	0.932*
	Neoplastic	7 (19.4)	19 (15.2)		
	Congenital	7 (19.4)	27 (21.6)		
	Degenerative	10 (27.8)	33 (26.4)		
	Others	1 (2.8)	7 (5.6)		
Anatomical classification, n (%)					
	Upper cervical	14 (38.9)	43 (34.4)	0.968	0.809*
	Mixed	6 (16.7)	16 (12.8)		
	Cervical-Thoracic	1 (2.8)	6 (4.8)		
	Subaxial	15 (41.7)	60 (48.0)		
Surgery type, n (%)					
	Elective	30 (83.3)	116 (92.8)	0.845	0.085†
	Urgency	6 (16.7)	9 (7.2)		

* P-value result of Fisher's exact test;

† P-value result of Pearson's χ^2^ test.

**Table 3:** Postoperative complications: clinical and surgical

Characteristics	Surgery type		χ^2^	p value*
	Elective (n=146)	Urgency (n=15)		
Patients with one or more clinical complications, n (%)				
Yes	3 (2.1)	4 (26.7)	28.2	0.001
No	143 (97.9)	11 (73.3)		
Patients with one or more surgical complications, n (%)				
Yes	27 (18.5)	3 (20.0)	0.245	0.887
No	119 (81.5)	12 (80.0)		

* P-value value of Fisher's exact test.

Note that 36 patients had a total of 50 complications.

**Table 4:** Association between surgery type and complications

Complications		n	(Surgeries)* %	(Complications)† %
Clinical		9	5.6	18.0
	Pneumonia	5	3.1	10.0
	Urinary tract infection	3	1.9	6.0
	Deep vein thrombosis	0	0.0	0.0
	Pulmonary thromboembolism	1	0.6	2.0
Surgical		41	25.5	82.0
	Site infection	10	6.2	20.0
	Hematoma	0	0.0	0.0
	Cerebrospinal fluid leak	0	0.0	0.0
	Dehiscence	2	1.2	4.0
	Neurological Impairment	3	1.9	6.0
	Early reoperation	3	1.9	6.0
	Late reoperation	7	4.3	14.0
	Instrument breakage	3	1.9	6.0
	Pseudoarthrosis	0	0.0	0.0
	Other	7	4.3	14.0
Death		6	3.7	12.0

* % in relation to all surgeries (n = 161);

† % in relation to all complications found (n=50).

Note – 36 patients had a total of 50 complications.

As table 4 shows, the most frequent complications were surgical site infections (n=10; 6.2%) and late reoperation for any reason (pseudoarthrosis, adjacent level disease, etc.) (n=7; 4.3%). The most common clinical complication was pneumonia, occurring in 3.1% (n=5) of the patients.

## DISCUSSION

Research addressing open PCSS complications is important, as this approach is often used in practice despite developments and advances in other techniques. Identifying factors associated with a high risk of complications is useful for predicting adverse outcomes and for patient pre- and postoperative counseling. We hypothesized that factors such as age, sex, urgency of surgery, and spinal segment could be associated with the development of complications. Current literature provides heterogeneous and sometimes conflicting evidence on risk factors, often owing to population differences.^(4-8)^ The present study highlighted the etiological differences between the sex and age of the patients who underwent PCSS. Most of the patients were men (65.4%), which is similar to the findings of a retrospective study by Harel et al., who reported that 72.8% of the patients were males.^(4)^ The mean age of the patients who underwent surgery in the present study was relatively low at 45.1 years. This may be because we had more trauma cases in our series compared with other studies. This thought is corroborated by other studies evaluating traumatic cervical spine surgery, which reported a predominance of men and a mean age of approximately 40.^(14,15)^ In contrast, a systematic review and meta-analysis of studies reporting data on degenerative cervical spine disease showed an average patient age of greater than 50.^(16)^ Harel et al.'s study revealed a mean age of greater than 50 in a population of patients in which degenerative cases represented a larger proportion than non-degenerative/trauma.^(4)^ Therefore, our findings showing a mean age of 43.7 years in trauma cases and 55.7 years in degenerative cases are consistent with those of other studies.^(14,15)^

Our results from the investigation of complication and death rates are in accordance with those of previous studies despite the differences in population characteristics between studies. Harel et al. discovered a complication rate of 20.3% in a cohort of 59 patients who underwent PCSS.^(4)^ Among the complications reported in our series, surgical site infection (6.2%) and late reoperation (4.3%) were the most common. Similarly, Lantz et al. investigated complications in 1593 patients who underwent posterior cervical surgery and found that 8.3% of the complications were infections and 9.3% were reoperations.^(17)^ Based on the higher prevalence of infections and reoperations found in Lantz et al.'s and the present study, these complications should be addressed and emphasized in preoperative patient counseling.

Few serious complications were reported in our study; these included worsening of urinary dysfunction, bilateral C5 paresis with progressive improvement during outpatient follow-up, and six deaths (3.7%) during hospitalization. Harel et al. reported a mortality rate of 2%. This comparatively low mortality rate can be explained by the fact that the present study had a higher prevalence of patients with cervical trauma, which corresponded to most of the patients who died.^(4)^ However, most of the reported complications were mild and transient (82%), suggesting the safety of the posterior surgical approach.

Despite the large number of patients evaluated in the present study, we did not identify any factors associated with the incidence of complications, except for the urgency of the procedure (elective vs. urgent), which tended to increase complications. This is not unexpected and likely not related to the surgical approach itself but to the patient's premorbid condition.

### Strengths and limitations

We described the characteristics and outcomes of a large cohort of patients who underwent open PCSS, providing insights into the factors associated with a higher risk of complications. This study was limited by its retrospective nature and heterogeneous follow-up period. In addition, this was a single-center study, and all surgeries were completed by a single surgeon.

## CONCLUSION

Posterior cervical spine surgery was more common in men, and patients with degenerative diseases had a higher average age. No statistically significant associations were revealed between complications and patient characteristics; however, our study revealed a trend towards more complications in emergency than elective surgeries. Serious complications are infrequent and less common in elective procedures; however, a risk of death, albeit low, has been reported, particularly in patients with cervical spine injury due to trauma. Understanding the epidemiology and complications is fundamental to prevention and important in providing appropriate advice prior to procedures.
